# Phytochemical Constituents and Toxicity of *Duguetia furfuracea* Hydroalcoholic Extract in *Drosophila melanogaster*

**DOI:** 10.1155/2014/838101

**Published:** 2014-11-10

**Authors:** Francisca Valéria Soares de Araújo Pinho, Gustavo Felipe da Silva, Giulianna Echeverria Macedo, Katiane Raquel Muller, Illana Kemmerich Martins, Ana Paula Lausmann Ternes, José Galberto Martins da Costa, Margareth Linde Athayde, Aline Augusti Boligon, Jean Paul Kamdem, Jeferson Luis Franco, Irwin Rose Alencar de Menezes, Thaís Posser

**Affiliations:** ^1^Departamento de Química Biológica, Universidade Regional do Cariri, 63100-000 Crato, CE, Brazil; ^2^Departamento de Química, Programa de Pós-Graduação em Bioquímica Toxicológica, Universidade Federal de Santa Maria, 97105-900 Santa Maria, RS, Brazil; ^3^Centro Interdisciplinar de Pesquisa em Biotecnologia, Universidade Federal do Pampa, Campus São Gabriel, 97300-000 São Gabriel, RS, Brazil; ^4^Departamento de Farmácia Industrial, Laboratório de Pesquisa em Fitoquímica, Universidade Federal de Santa Maria, Prédio 26, Sala 1115, 97105-900 Santa Maria, RS, Brazil; ^5^Departamento de Bioquímica, Instituto de Ciências Básica da Saúde, Universidade Federal do Rio Grande do Sul, 90035-003 Porto Alegre, RS, Brazil

## Abstract

*Duguetia furfuracea* is frequently used as a medicinal plant in Brazil. However, studies have evidenced its cytotoxic, bactericide, and antitumor activities. In the present study we aimed to evaluate the potential toxicity of hydroalcoholic leaves extracts of *D. furfuracea* (HEDF) in a *Drosophila melanogaster* model. Toxicity was assessed as changes in locomotor performance, mitochondrial activity, oxidative stress, MAPKs phosphorylation, and apoptosis induction after exposure to HEDF concentrations (1–50 mg/mL) for 7 days. The phytoconstituents of the plant were screened for the presence of alkaloids, tannins, xanthones, chalcones, flavonoids, aurones, and phenolic acids. Exposure of adult flies to HEDF caused mitochondrial dysfunction, overproduction of ROS, and alterations in the activity of detoxifying enzymes GST, SOD and CAT. Induction of ERK phosphorylation and PARP cleavage was also observed, indicating occurrence of HEDF-induced cell stress and apoptotic cell death. In parallel, alterations in cholinesterase activity and impairments in negative geotaxis behavior were observed. Our study draws attention to the indiscriminate use of this plant by population and suggests oxidative stress as a major mechanism underlying its toxicity.

## 1. Introduction

The use of plant extracts in the treatment and/or prevention of various diseases including cancer and cardiovascular diseases is recognized since ancient time, and some of these plants have led to the development of an impressive number of new drugs [1, 2]. The growing use of plant extracts instead of synthetic compounds is primary because they are generally regarded as safe, easily accessible, affordable, and culturally acceptable form of health care trusted by large number of people [3–6]. Despite the beneficial effects of plant extracts, there are substantial evidences suggesting they can cause cytotoxicity [7, 8]. Therefore, evaluation of the toxic effects of plant extracts used in folk medicine seems to be imperative since they are generally consumed by population without concerns on their toxicity [9].

Oxidative stress, caused by overproduction of free radicals and/or alterations in antioxidant defense systems, is implicated as a mechanism of toxicity of many synthetic and natural compounds [10]. The antioxidant cellular defense system including enzymatic (glutathione-S-transferase (GST), catalase (CAT), and superoxide dismutase (SOD)) and nonenzymatic (glutathione (GSH), ascorbic acid (vitamin C), *α*-tocopherol (vitamin E), and *β*-carotene (vitamin A)) antioxidants act on reactive oxygen species (ROS) resulting from physiological or pathological processes [11]. Environmental stressors, toxic agents, and natural compounds as flavonoids [12–14] are reported to modulate the phosphorylation of mitogen activated protein kinases family (MAPKs), represented by ERK, c-Jun NH_2_-terminal kinase (JNK), and p38^MAPK^ kinases, which participate in signaling pathways that are responsible for many cellular functions, such as growth, differentiation, and apoptosis.

*Duguetia furfuracea*, belonging to the Annonaceae family, is a perennial and shrubby species found in the Central West, Southeast, and Northeast regions of Brazil [15, 16]. It is popularly known as “Araticum-Miúdo,” “Araticum-seco,” and “Ata brava” and is used in Brazilian folk medicine as an antihyperlipidemic and anorectic agent [17]. Accordingly,* D. furfuracea* has also been reported in the treatment of rheumatism and renal colic [18], as an antiparasitary agent. The powder of its seeds is used in the treatment of pediculosis [19].* D. furfuracea* has shown to exhibit larvicidal activity against* Aedes aegypti* [20], and isolated alkaloids from the stem bark of the plant have been reported to exhibit antitumoral, trypanocidal and leishmanicidal activities [21]. Although* D. furfuracea* have been used by population due its therapeutic properties, recently, attention has been paid regarding the toxicity of this plant species. The aqueous extract of the leaves of* D. furfuracea* presented toxic effect in pregnant rats [22]. Studies have demonstrated cytotoxic effects of the leaves of* D. furfuracea* in bacteria and animal models [23, 24].

Phytochemical analysis of essential oil from leaves of* D. furfuracea* revealed the presence of sesquiterpenoids [25] and the bark of the underground stem revealed the presence of the alkaloid, (-)-duguetine *β*-N-oxide [26]; in addition, flavonoids and several alkaloids [16] have been also described for aerial parts of* Duguetia furfuracea.* Previous studies demonstrated the at least five alkaloids isolated from this plant have cytotoxic, antitumoral, trypanocidal, and leishmanicidal activities [21], while sesquiterpenes are potential anticancer agents [27]. Flavonoids are recognized by their beneficial and prooxidative effects, depending on the concentration and frequency of exposure, presenting properties such as anti-inflammatory, diuretic, antimicrobial, antiviral, antioxidant, and proapoptotic [12].

In the present study, we investigated the potential toxicity of a hydroalcoholic extract from leaves of* D. furfuracea* (HEDF) in a fruit fly* D. melanogaster* model. The advantage of using this model is based on the fact that it raises few ethical concerns and has served as a unique and powerful model to study human genetics, diseases and for screening synthetic and natural compounds [28, 29]. Particularly, we investigated the behavioral (negative geotaxis assay and acetylcholinesterase activity) and biochemical markers of oxidative stress and apoptotic cell death (ROS generation, cell viability, antioxidant enzymes, p38^MAPK^ and ERK phosphorylation, and PARP cleavage) following exposure of flies to HEDF up to seven days. In addition, the identification and quantification of phenolic compounds present in HEDF were carried out by HPLC.

## 2. Materials and Methods

### 2.1. Materials

Reduced glutathione (GSH), tetramethylethylenediamine (TEMED), sodiumorthovanadate (Na_3_VO_4_), Quercetin (Q4951), 3-(4,5-dimethylthiazol-2-yl)-2,5-diphenyltetrazolium bromide (MTT), Secondary antibodies (anti-rabbit IgG) horse radish peroxidase (HRP) conjugated, 5,5-dithiobis (2-nitrobenzoic acid) DTNB (D8130), acetylthiocholine iodide (A5751), 1-Chloro, 2,4-dinitrobenzene (CDNB) (237329), and 2′,7′-dichlorofluorescein diacetate (DCFH-DA) (35845) were obtained from Sigma-Aldrich (St. Louis, MO). The anti-phospho-p38 (Thr180/Tyr182), total anti-p38, anti-phospho ERK1/2 (Thr202/Tyr204), and anti-total-ERK1/2 antibodies and b-actin were purchased from Cell Signaling (Beverly, MA, USA). Poly (ADP) ribose polymerase (PARP) antibody was obtained from Santa Cruz Biotechnology (Santa Cruz, CA). SDS acrylamide,* bis*-acrylamide chloride, hybond nitrocellulose, and dithiothreitol (DTT) were obtained from GE Healthcare Life Division. Acetonitrile and formic, gallic, chlorogenic, ellagic, and caffeic acids were purchased from Merck (Darmstadt, Germany). Catechin, quercetin, quercitrin, isoquercitrin, rutin, and kaempferol were purchased from Sigma Chemical Co. (St. Louis, MO, USA). High performance liquid chromatography (HPLC-DAD) was performed using HPLC system (Shimadzu, Kyoto, Japan) prominence autosampler (SIL-20A) equipped with plunger pumps LC-20AT Shimadzu DGU connected to the integrator with 20A5 Degasser CBM 20A UV-VIS detector DAD (diode) and SPD-M20A LC 1.22 SP1 software solution. All other chemicals were of analytical grade.

### 2.2. Plant Material

Leaves of* D. furfuracea* were collected from Barreiro Grande, Crato-Ceará (7°22′2.8′′S, 39°28′42.4′′W and altitude of 892 m above sea level), Brazil, in September 2011, and identified by Dr. Maria Arlene Pessoa da Silva. A voucher specimen (n. 6703) was deposited in the Herbarium Caririense Dárdano de Andrade Lima (HCDAL) of the Regional University of Cariri (URCA).

### 2.3. Preparation of Crude Ethanolic Extract

Fresh leaves (1,050 g) of* D. furfuracea* were washed with water, then crushed, and macerated with 99.8% of ethanol and water (1 : 1, v/v) for three days. The suspension was filtered, and the solvent evaporated under reduced pressure and lyophilized to obtain 261.13 g of crude ethanolic extract. The dried extract was then kept frozen prior to use.

### 2.4. Preliminary Phytochemical Analysis

Classes of secondary metabolites were screened for the presence or absence of alkaloids, tannins, xanthones, chalcones, flavonoids, and aurones in HEDF, using the methods previously described by Matos [30]. This is a qualitative test based on visual observation of change in coloration or precipitate formation in addition to specific reagents.

### 2.5. Identification and Quantitation of Phenolic Compounds of HEDF by HPLC

Reverse phase chromatographic analyses were carried out under gradient conditions using C_18 _column (4.6 mm × 250 mm) packed with 5 *μ*m diameter particles. The mobile phase was water containing 1% formic acid (A) and acetonitrile (B), and the composition gradient was 13% of B until 10 min and changed to obtain 20%, 30%, 50%, 60%, 70%, 20%, and 10% B at 20, 30, 40, 50, 60, 70, and 80 min, respectively [31] with slight modifications. HEDF was analyzed at a concentration of 5 mg/mL. The presence of ten antioxidants compounds, namely, gallic, chlorogenic, ellagic, and caffeic acids, catechin, quercetin, quercitrin, isoquercitrin, rutin, and kaempferol were investigated. The identification of these compounds was performed by comparing their retention time and UV absorption spectrum with those of the commercial standards. The flow rate was 0.7 mL/min, injection volume 40 *μ*L and the wavelength 254 nm for gallic acid, 280 nm for catechin, 325 nm for caffeic, ellagic, and chlorogenic acids, and 365 nm for quercetin, isoquercitrin, quercitrin, rutin, and kaempferol. All the samples and mobile phase were filtered through 0.45 *μ*m membrane filter (Millipore) and then degassed by ultrasonic bath prior to use. Stock solutions of standards references were prepared in the HPLC mobile phase at a concentration range of 0.030–0.250 mg/mL for kaempferol, quercetin, quercitrin, isoquercitrin, catechin, and rutin and 0.030–0.250 mg/mL for gallic, caffeic, ellagic, and chlorogenic acids. All chromatography operations were carried out at ambient temperature and in triplicate. The limit of detection (LOD) and limit of quantification (LOQ) were calculated based on the standard deviations of the responses and the slopes using three independent analytical curves, as defined by [32]. LOD and LOQ were calculated as 3.3 and 10 σ/*S*, respectively, where σ is the standard deviation of the response and* S* is the slope of the calibration curve.

### 2.6. *Drosophila melanogaster* Stock and Media

*Drosophila melanogaster* (Harwich strain) was obtained from the National Species Stock Center, Bowling Green, OH, USA. The flies were maintained at 25 ± 1°C and 60–70% relative humidity. The diet was composed of 6 mL of cereal flour, corn flour, water, and antifungal agent (Nipagin) as previously described [14].

### 2.7. Experimental Procedure—Flies Exposed to HEDF

#### 2.7.1. Mortality

In order to determine the doses and the duration of exposure, 45 adults flies of both genders (1- to 5-day-old) per vial were exposed for 7 days to various concentrations of HEDF (0, 1, 2, 10, 20, 50, 100, and 200 mg/mL) mixed to the diet. Each concentration contained three replicates. The number of dead and alive flies was recorded daily for 7 days. Based on these preliminary data, the concentrations range of 0, 1, 10, and 50 mg/mL of HEDF were chosen with 7-day-exposure.

#### 2.7.2. Negative Geotaxis Test

Locomotor activity of flies was determined based on negative geotaxis behavior assay as previously described [33] with some modifications. The flies were exposed to HEDF as described above. Following exposure, 10 flies (both genders) were sorted under a brief ice anesthesia and transferred in labeled vertical glass columns tube (15 cm length and 1.5 cm in diameter). After 30 min of recovery from ice, flies were gently tapped to the bottom of the tube, and the number of flies that climbed up to 6 cm mark of the column (i.e., the top) in 5 sec as well as those that remained below the mark was counted separately. The procedure was repeated five times per group at 1 min intervals.

#### 2.7.3. Mitochondrial Activity-MTT Reduction Assay

Cell viability was determined by the mitochondrial dehydrogenase activity using the 3-(4,5-dimethylthiazol-2-yl)-2,5-diphenyltetrazolium bromide (MTT). After exposure, the viability of HEDF-treated and nontreated flies was evaluated in the whole body of flies according to the method described by Sudati et al. [34]. For this set of experiment, three whole flies of similar size were used per well. The absorbance was measured at 540 nm in a microplate reader (2,300 Enspire Multilabel Plate Reader, 2009), and the results were expressed as mean of absorbance of three determinations performed in triplicate.

#### 2.7.4. Measurement of ROS Production

ROS generation was measured as a general index of oxidative stress using the 2′,7′-dichlorofluorescein diacetate (DCHF-DA), a nonpolar compound, which after conversion to a polar derivative by intracellular esterase activity rapidly reacts with ROS to form the highly fluorescent compound dichlorofluorescein (DCF) [35, 36]. Briefly, after exposure of flies to HEDF (0–50 mg/mL), the whole body of 20 flies was homogenated in 1 mL of 20 mM Tris buffer (pH 7.0) and then centrifuged at 1.000 rpm for 10 min at 4°C. An aliquot of 20 *μ*L of the supernatant was incubated with 6 *μ*L of 10 mM DCFH-DA for 60 min. The formation of the fluorescent product DCF was measured using a microplate reader (2300 Multilabel Plate reader Enspire, 2009) at 488 and 525 nm, excitation and emission wavelengths, respectively. The results were expressed in arbitrary units as the mean of DCF fluorescence.

*(1) Estimation of Protein Thiol (PSH) and Nonprotein Thiol (NPSH)*. Protein thiols (PSH) and nonprotein (NPSH) were measured as previously described [37]. For thiols estimation, 20 flies were homogenated in 300 *μ*L of percholic acid (PCA; 0.5 M) and centrifuged at 10.000 ×g for 5 min at 4°C. The supernatant was used to measure NPSH and the pellet was resuspended in 200 *μ*L of Tris/HCl 0.5 M, pH 8.0, to measure PSH.

*(2) Activity of Selected Enzymes (AChE, CAT, GST, and SOD)*. To determine acetylcholinesterase (AchE), catalase (CAT), glutathione-S-transferase (GST), and superoxide dismutase (SOD) activities, flies homogenates were centrifuged at 1.000 ×g for 5 min at 4°C, and an aliquot of the supernatant (S1) was used for measurement of AchE [38], while the (S1) was centrifuged again at 14.000 ×g for 30 min at 4°C, for the measurement CAT [35], GST [36] and SOD [39].

#### 2.7.5. Western Blotting

Western blotting methodology was performed according to the method described [14]. Forty flies from HEDF-treated and untreated were homogenized at 4°C in 200 *μ*L of buffer (pH 7.0) containing 50 mM Tris, 1 mM EDTA, 0.1 mM phenyl methyl sulfonyl fluoride, 20 mM Na_3_VO_4_, 100 mM sodium fluoride, and phosphatase inhibitor cocktail. The homogenates were centrifuged at 1.000 ×g for 10 min at 4°C and the supernatants (S1) were collected. After determination of the protein [40], 10% DTT was added to the samples. Then, the samples were frozen at −20°C for later determination of total and phosphorylated forms of ERK 1/2 and phosphorylated form of p38^MAPK^ using specific antibodies. The immunoblots were visualized and quantified on the 400 MM Pro Bruker imaging system using ECL detection reagents. The density of the bands was measured and expressed as percent of increase over the control (nontreated flies). *β*-actin, total p38^MAPK^ and total ERK were used as loading controls for Western blotting experiments. Protein levels were quantified using bovine serum albumin as standard.

### 2.8. Statistical Analysis

The results are shown as mean ± SEM (standard error of mean) of three independent experiments performed in duplicate. Statistical significance was measured by one-way analysis of variance (ANOVA), followed by Dunnett's or Tukey's posttest when appropriated. Differences between groups were considered to be significant when *P* < 0.05.

## 3. Results

### 3.1. Phytochemical Screening of HEDF

The presence of alkaloids, tannins, flavones, flavonols, chantonas, chauconas, and auronas was identified in HEDF (data not shown).

### 3.2. Identification and Quantification of Phenolic Compounds of HEDF by HPLC

**Figure 1:**
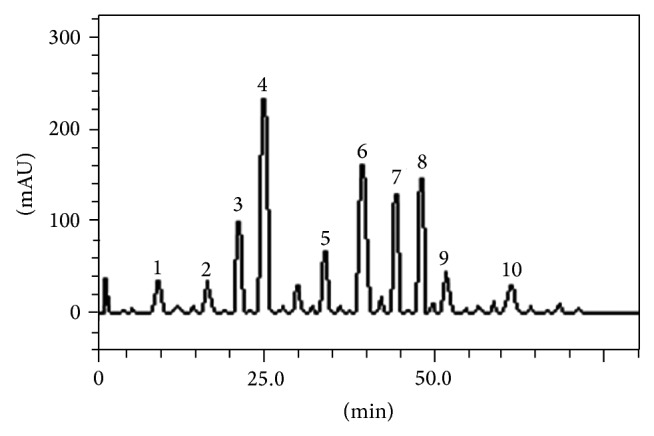
High performance liquid chromatography profile of HEDF, detection UV, was at 325 nm. Gallic acid (peak 1), catechin (peak 2), chlorogenic acid (peak 3), caffeic acid (peak 4), ellagic acid (peak 5), rutin (peak 6), isoquercitrin (peak 7), quercitrin (peak 8), quercetin (peak 9), and kaempferol (peak 10). Calibration curve for gallic acid:* Y* = 14286*x* + 1395.8 (*r* = 0.9996); catechin:* Y* = 15097*x* + 1189.3 (*r* = 0.9997); caffeic acid:* Y* = 12758*x* + 1259.7 (*r* = 0.9996); chlorogenic acid:* Y* = 13461*x* + 1275.3 (*r* = 0.9992); ellagic acid:* Y* = 13576*x* + 1346.4 (*r* = 0.9999); rutin:* Y* = 12845 + 1305.7 (*r* = 0.9999); quercetin:* Y* = 13560*x* + 1192.6 (*r* = 0.9991), isoquercitrin:* Y* = 12873*x* + 1325.6 (*r* = 0.9998); quercitrin:* Y* = 11870*x* + 1329.8 (*r* = 0.9993); and kaempferol:* Y* = 14253*x* + 1238.9 (*r* = 0.9997).

HPLC fingerprinting of HEDF revealed the presence of the gallic acid (*t*_*R*_ = 9.95 min; peak 1), catechin (*t*_*R*_ = 16.08 min; peak 2); chlorogenic acid (*t*_*R*_ = 20.14 min; peak 3), caffeic acid (*t*_*R*_ = 24.63 min; peak 4), ellagic acid (*t*_*R*_ = 37.29 min; peak 5), rutin (*t*_*R*_ = 39.87 min; peak 6), isoquercitrin (*t*_*R*_ = 44.93 min; peak 7), quercitrin (*t*_*R*_ = 48.15 min; peak 8), quercetin (*t*_*R*_ = 51.07 min; peak 9), and kaempferol (*t*_*R*_ = 61.56 min; peak 10). They were identified by comparisons with the retention times and UV spectra of the standards analyzed under the same analytical conditions (Figure 1). Caffeic acid (33.17 ± 0.03 mg/g), rutin (20.05 ± 0.01 mg/g), quercitrin (19.07 ± 0.02 mg/mL), and isoquercitrin (18.61 ± 0.01 mg/mL) were the major components present in HEDF, while kaempferol (5.36 ± 0.01 mg/mL), catechin (5.31 ± 0.01 mg/mL) and gallic acid (5.29 ± 0.01 mg/mL) were less abundant (Table 1).

### 3.3. Effects of HEDF Exposure on Survival of Flies

**Figure 2:**
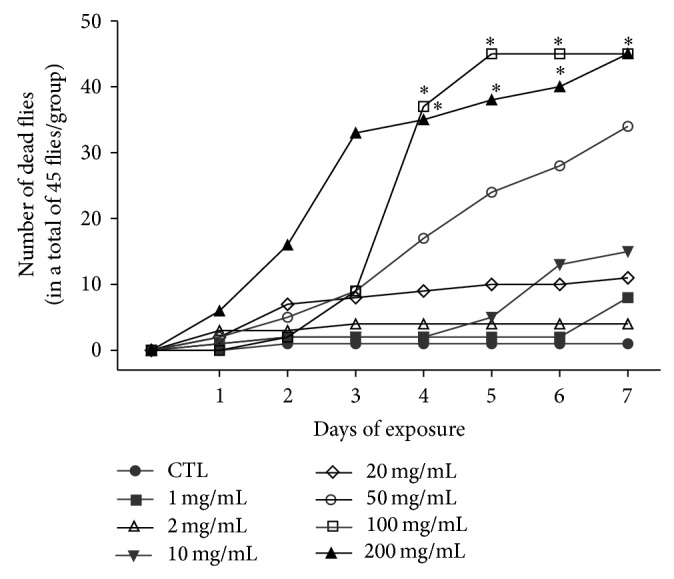
Mortality curve of HEDF (0–200 mg/mL) exposed flies. The number of live and dead flies was counted daily for seven days. The data represent the mean ± SEM of three separate experiments and are expressed number of dead flies in relation to total number of flies. Doses of 100 and 200 mg/mL were significant when compared to control group ^*^ *P* < 0.05.

Our preliminary results showed that exposure of flies to HEDF (0–200 mg/mL) caused 78% (*P* < 0.05) mortality at concentration of 50 mg/mL after 7 days of exposure, while 100 and 200 mg/mL of HEDF caused 100% mortality (Figure 2). Considering the high toxicity of the higher concentrations of the extract, we decided to use the concentrations of 1, 10, and 50 mg/mL of HEDF for the evaluation of biochemical analyses.

#### 3.3.1. HEDF Exposure Induced Locomotor Deficits in* D. melanogaster*

**Figure 3:**
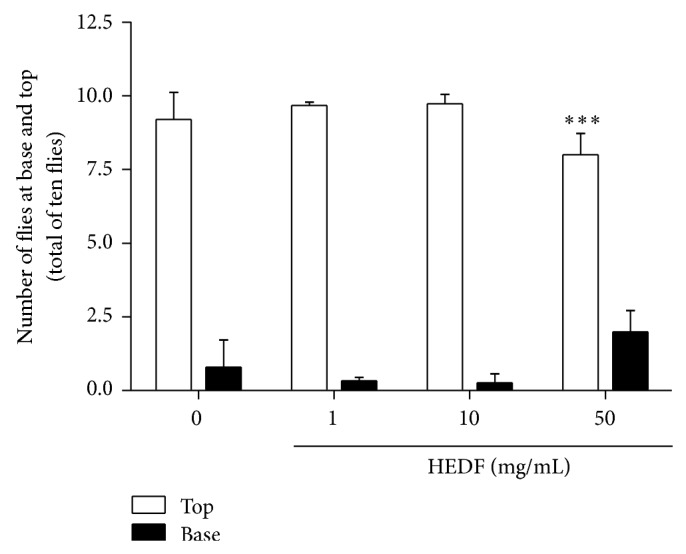
Locomotive performance in groups of 10 flies exposed to HEDF for seven days at doses of 0, 1, 10, and 50 mg/mL by negative geotaxis test. The measurements were repeated five times at intervals of 1 min. The test was performed in triplicate (*n* = 10), and data represents the mean ± SEM of the number of flies that were in the top and bottom. Exposure of flies to 50 mg/mL of HEDF caused significant deficit in locomotive performance when compared to control group (^***^ *P* < 0.0001).

**Figure 4:**
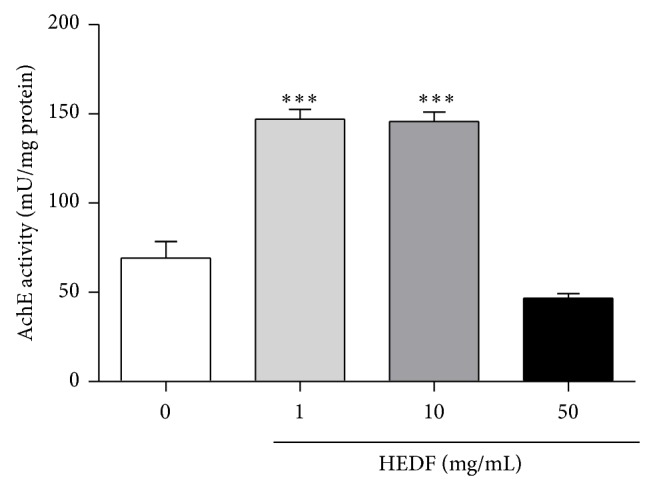
Effect of HEDF exposed flies on the activity of AchE. HEDF (1 and 10 mg/mL) induced alteration in the activity of AchE in HEDF-treated flies after 7 days of exposure. The data represent the mean ± SEM of three replicates performed in duplicate. ^***^ *P* < 0.001 and ^*^ *P* < 0.05 indicate significant difference when compared to control.

Exposure of flies to 1 and 10 mg/mL of HEDF for 7 days did not change the locomotion activity of flies when compared to control (Figure 3, *P* > 0.05). However, flies treated with 50 mg/mL of HEDF remained mostly at the bottom of the column when compared to the control group (Figure 3) indicating locomotor deficit. In parallel to behavioral parameter, the activity of AchE was evaluated since acetylcholine is the main excitatory neurotransmitter of the insect central nervous system [41]. As shown in Figure 4, the activity of AchE was significantly elevated (*P* < 0.001) in flies exposed to 1 and 10 mg/mL of HEDF, when compared to control flies. In contrast, 50 mg/mL of HEDF caused a significant inhibition of AchE activity when compared to control flies (Figure 4, *P* < 0.05).

#### 3.3.2. Mitochondrial Activity of HEDF Exposed Flies

**Figure 5:**
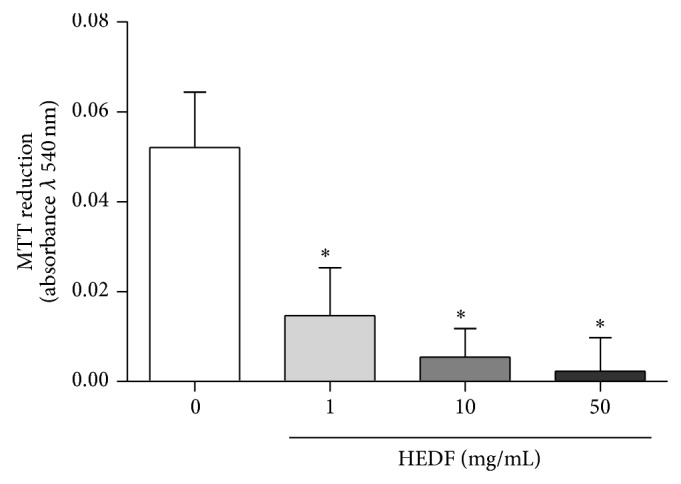
MTT reduction of flies exposed to different concentrations of HEDF for 7 days. The experiments were performed in triplicate, and the bars represent an average ± SEM of absorbance obtained in control group (untreated flies) and HEDF exposed flies. *n* = 10, ^*^ *P* < 0.05 versus control flies (untreated).

The metabolic viability of cells was evaluated by assessing the MTT reducing capacity of flies' homogenates. HEDF exposure caused a significant decrease in MTT reduction at concentrations of 10 and 50 mg/mL when compared to control flies (Figure 5) (*P* < 0.05).

#### 3.3.3. Oxidative Stress Analysis

**Figure 6:**
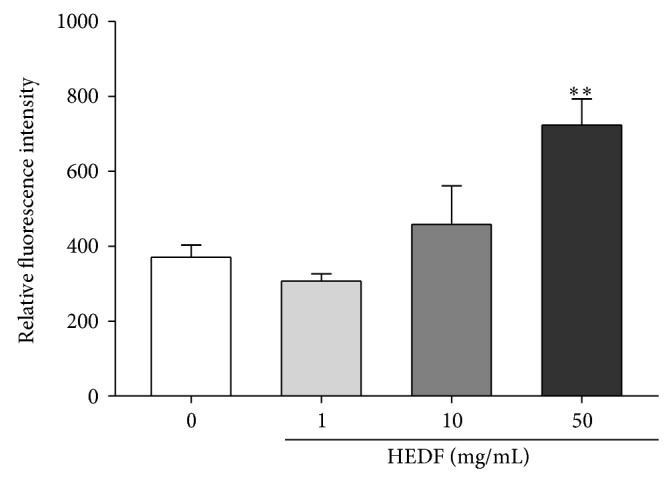
ROS production in response to exposure to HEDF (1–50 mg/mL) flies for seven days. ROS production is represented by the intensity of fluorescence emitted by the DCF and represents the mean ± SEM of three independent experiments, performed in triplicate (*n* = 3). ^**^ *P* < 0.01 compared to the control group (untreated flies).

**Figure 7:**
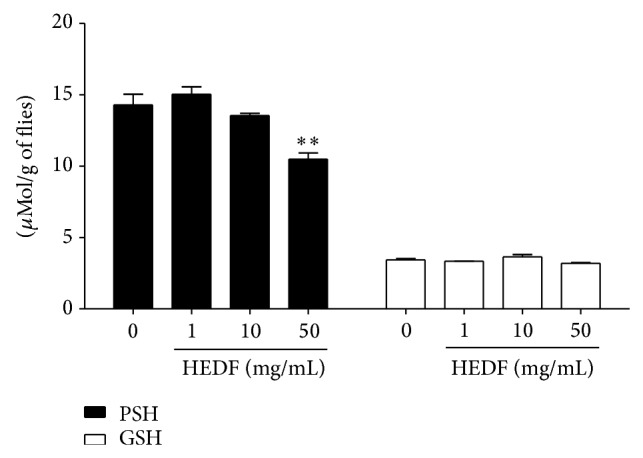
Levels of protein (PSH) and nonprotein thiol (NPSH) in homogenates of flies exposed to different concentrations of HEDF (1, 10 and 50 mg/mL) for 7 days. There was a significant decrease in PSH levels at dose of 50 mg/mL of HEDF. PSH or NPSH levels are represented in *μ*Mol/g of flies. ^**^ *P* < 0.01 versus untreated flies. Data are expressed as mean ± SEM of *n* = 4 independent experiments, performed in triplicate.

As depicted in Figure 6, HEDF (50 mg/mL) caused significant increase of DCF fluorescence in the homogenates of flies when compared to control (*P* < 0.01). Our results also revealed that only the highest concentration of HEDF (50 mg/mL) exposed flies caused a significant decrease in the levels of nonprotein thiols (NPSH) (Figure 7, *P* < 0.05). However, no changes in the levels of protein thiols (PSH) were observed in HEDF exposed flies when compared to control (Figure 7).

**Figure 8:**
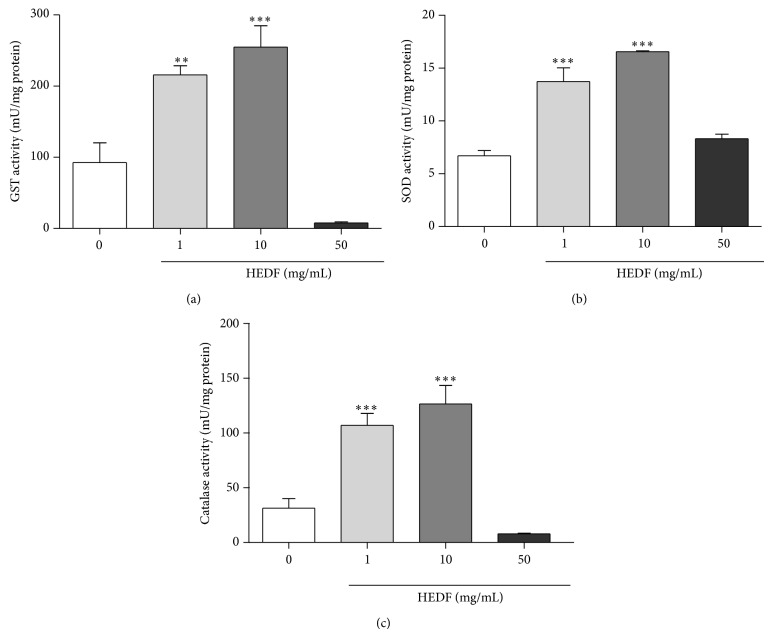
Analysis of antioxidant enzymes activities after 7 days exposure of flies to various concentrations of HEDF (1–50 mg/mL). (a) GST, (b) SOD, and (c) CAT activities are expressed as mU/mg of protein. The data represent the mean ± SEM of three independent replicates performed in duplicates. ^*^ *P* < 0.05, ^**^ *P* < 0.01, and ^***^ *P* < 0.001 versus control flies (untreated).

Considering that oxidative stress was induced by exposure of flies to HEDF (evidenced by increased ROS generation and decreased NPSH levels), we measured the activity of GST, SOD, and CAT, which are antioxidant enzymes involved in the cell adaptive response to oxidative stress. HEDF induced a marked increase in the activities of GST (Figure 8(a)), SOD (Figure 8(b)) and CAT (Figure 8(c)) at concentrations of 1 and 10 mg/mL when compared to their respective control (*P* < 0.001). However, at the highest concentration tested (50 mg/mL), there was a substantial decrease in GST activity (Figure 8(a), *P* < 0.05), while CAT and SOD activities did not change (Figures 8(b) and 8(c)).

#### 3.3.4. Phosphorylation of MAPKs and PARP Cleavage

**Figure 9:**
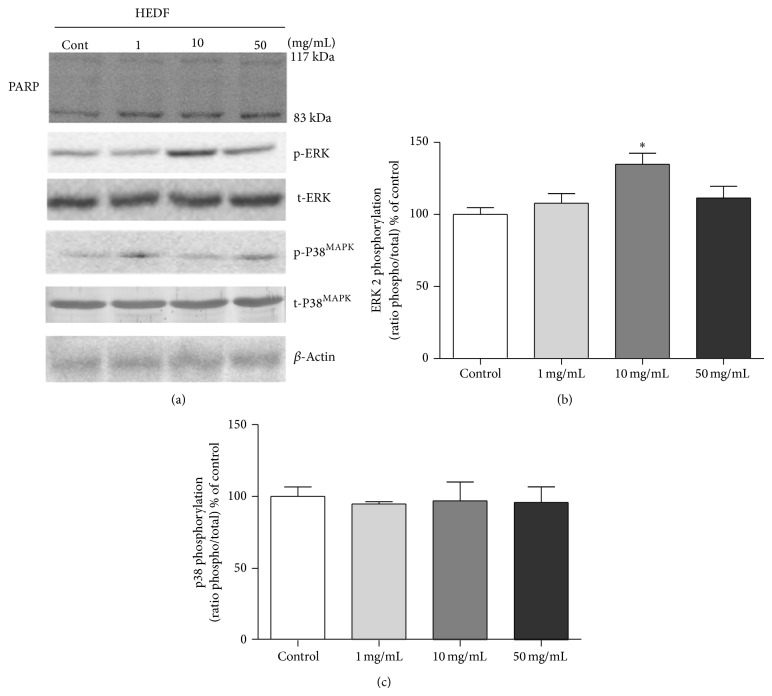
Western blot analysis of the phosphorylated and total forms of ERK (a) and p38^MAPK^ (b) and PARP cleavage in homogenates of flies exposed to HEDF (1–50 mg/mL) for seven days. The total content and phosphorylation of proteins were detected by specific antibodies and the reaction was developed by the method of detection by enhanced chemiluminescence (ECL). The graph represents the quantification of the immunoreactive bands and means ± SEM of three independent experiments (% of control) (*n* = 3) ^*^ *P* < 0.05 and ^**^ *P* < 0.01 compared to the control group.

The phosphorylation of MAPK family components was assayed after 7 day-exposure of* D. melanogaster* to HEDF. Phosphorylation of ERK2 was significantly increased in flies treated with 10 mg/mL of HEDF, while p38^MAPK^ was not modified at all the concentrations tested when compared to untreated flies (Figures 9(b) and 9(c)). Exposure to all concentrations of extract caused PARP cleavage (Figure 9(a)), indicating the occurrence of apoptotic cell death.

## 4. Discussion

Although the use of plant extracts has been reported to exert a variety of pharmacological actions by different mechanisms, there is evidence that some of them can cause toxicity [42, 43]. Therefore, it is imperative to explore the toxicity potential of plant extracts popularly used in folk medicine. In the present study, we investigated the potential toxicity of the hydroalcoholic extract of the leaves of* D. furfuracea* (HEDF) in a* Drosophila melanogaster* model and identified some phytochemicals of the plant extract. Our results demonstrated that 7 days of exposure of* D. melanogaster* to HEDF caused toxicity in a process involving oxidative stress, alterations in the antioxidant enzymes, MAPK proteins phosphorylation, and apoptotic cell death.

The flies exposed to HEDF showed a significant decrease in the MTT reduction, indicating that viability of flies' cells was compromised. In the same line, flies that were exposed to HEDF revealed a significant increase in ROS production at concentration of 50 mg/mL, whereas lower concentrations of HEDF (1 and 10 mg/mL) seemed to stimulate ROS generation in minor extent and improve antioxidant enzymes activities. In parallel, PARP cleavage, a general index of apoptotic cell death, was observed at all concentrations analyzed. The observed increase of antioxidant enzymes could represent an adaptive cellular response, in counteracting HEDF toxicity. This apparent adaptive response was possibly induced by the mild increase of ROS at the lower concentrations of HEDF, leading to an increase of GST, SOD, and CAT activities in the flies, thus improving its antioxidant capacity. The highest level of ROS generation in the homogenates of flies exposed to 50 mg/mL may be at least, in part, responsible for the depletion of GST and CAT activities, decrease in mitochondrial activity, as well as NPSH oxidation. In fact, oxidative stress results from an unbalance between the production of reactive species and the capacity of cellular antioxidant defense to neutralize those species. When this capacity is overwhelmed, it leads to a diversity of cellular damages [44]. A significant reduction of NPSH (i.e., GSH) levels was identified in flies exposed to the 50 mg/mL of HEDF. It is well described that low levels of cellular GSH results from an increase in oxidative stress associated with toxic agents (ROS inducers) [45, 46]. It is interesting to note that GST, SOD, and CAT activities were increased in the flies that were exposed to the lower doses of HEDF (1 and 10 mg/mL). Low to moderate level of oxidative stress has been associated with increased antioxidant defense system [46]. In previous studies, flies exposed to rotenone presented a significant elevation of antioxidant enzymes, and this effect was related to the increased ROS generation and formation of toxic aldehydes [47]. Excessive ROS generation can cause lipid peroxidation, mitochondrial dysfunction, and damage to proteins, lipids, and nucleic acids, thereby, altering the normal function of the cells [48].

The activity of acetylcholinesterase (AChE) was increased in the flies exposed to 1 and 10 mg/mL of HEDF and was inhibited at 50 mg/mL. In fact, acetylcholine (Ach) plays several functions in the nervous system, acting in cognitive process, motivation and reward, stimuli processing, and the sleep cycle. The inhibition of AChE compromises the hydrolysis of the neurotransmitter ACh, leading to the accumulation of this neurotransmitter in the synapses [49, 50]. Many studies with toxic agents, such as rotenone, paraquat, and organophosphorus, revealed that these agents can lead to the inhibition of AchE, generating the loss of the cholinergic homeostasis, which causes many neurochemical and neurobehavioral disturbances [51–53]. The central nervous system is very susceptible to oxidative stress due to high oxygen consumption, lower levels of antioxidant defenses, high levels of polyunsaturated fatty acids (phospholipid membrane), and high levels of iron [54]. The inhibition of AchE at 50 mg/mL of HEDF correlated well with the observed locomotor deficits induced by HEDF at the same concentration. These results indicate that HEDF-induced toxicity may be associated with impairment of neurological functions. There is evidence that enhanced activation of AchE can cause a reduction of cholinergic neurotransmission and affect other related functions including cell proliferation and promote apoptosis [55, 56]. In the present study, exposure of flies to 1 and 10 mg/mL of HEDF significantly increased AchE activity. Alterations in AchE activity can compromise the normal motor activity of the flies, and this was evidenced in our study by the impairment of locomotive performance in the negative geotaxis behavior assay.

Mitogen-activated protein kinases (MAPKs) are a family of proteins that participate in transduction pathways affected by various environmental pollutants, including heavy metals [14]. Usually, the ERK is activated by mitogenic stimuli and regulates mainly the growth and differentiation. The JNK and the p38^MAPK^ are activated by cellular stress and inflammatory cytokines and are involved in the apoptotic process. These proteins regulate the transmission of signals from membrane to the nucleus and are well conserved from unicellular to more complexes organisms [57, 58]. Studies demonstrated that plant derivatives such as curcumin can modulate this pathway, mediating the antitumoral property attributed to this compound [58, 59]. In our study, exposure of flies to 10 mg/mL of HEDF significantly increased ERK phosphorylation. It well known that ERK pathway is susceptible to oxidative stress. In addition, hydrogen peroxide, metals, and other cell stressors are demonstrated to stimulate phosphorylation of this MAPK protein [58, 60], thus contributing to activation of several downstream targets as transcriptional factors. It was demonstrated that medicinal plants are able to induce gene activation dependent of the transcriptional factor Nrf-2 via ERK [61]. This transcription factor is known as the main regulator of the antioxidant cell response and control the expression of several antioxidant enzymes [61]. Here, the phosphorylation of ERK occurred in parallel with an increase in antioxidant enzymes activity, possibly in response to extracellular stimuli [62] induced by HEDF exposure. A possible link between the activity of these enzymes and ERK activation in our model must be further investigated.

In the present work, the phytochemical prospection and HPLC analysis of the leaves of HEDF revealed the presence of alkaloids and chalcones, in addition to polyphenolic compounds. Of particular importance, chalcone and some chalcone analogues have been reported to be toxic in zebrafish model [63]. Additionally, isolated oxoaporphine alkaloids from three species of the Annonaceae family, including* D. furfuracea*, have shown cytotoxic effect in the lineage of Hep2-cells (laryngeal carcinoma) [64]. On the other hand, Li et al., 2014 [65], showed the potentiality of total flavonoids from* Arachniodes exilis* to induce apoptotic cell death in human hepatoma HepG2 cells and cause oxidative stress. Similarly, it was shown that the flavonoid quercetin caused apoptotic cell death in different human tumoral cell types dependently of ERK phosphorylation and this effect occurred in parallel with a time dependent ROS production [12]. It was previously demonstrated that the flavonoid quercetin was protective against H_2_O_2_ induced toxicity only at low concentrations, while at higher concentration it inducted apoptotic cell death in hepatoma cells [66] and acting as a prooxidant agent [66, 67]. In accordance, our data shows that effects of* D. furfuracea* on survival and biochemical parameters in flies are related with the concentrations and time of exposure.

## 5. Conclusions

The present study demonstrated for the first time the toxicity of* Duguetia furfuracea* in the* Drosophila melanogaster* model system. The adverse effects of HEDF to flies were evidenced by alterations in several markers of cell stress and neurobehavioral parameters. The toxicity induced by the extract to flies may be attributed to an individual or synergistic action of phytochemicals found in this plant over the period of exposure. Overall, our results suggest that oxidative stress may be major mechanism underlying* D. furfuracea* induced toxicity in* D. melanogaster*.

## Figures and Tables

**Table 1:** Quantification of phenolic compounds from the HEDF.

Compounds	HEDF		LOD	LOQ
	mg/g	%	*μ*g/mL	*μ*g/mL
Gallic acid	5.29 ± 0.01^a^	0.52	0.015	0.049
Catechin	5.31 ± 0.01^a^	0.53	0.032	0.105
Chlorogenic acid	16.03 ± 0.02^b^	1.60	0.009	0.029
Caffeic acid	33.17 ± 0.03^c^	3.31	0.024	0.078
Ellagic acid	7.30 ± 0.01^d^	0.73	0.013	0.042
Rutin	20.05 ± 0.01^e^	2.00	0.027	0.090
Isoquercitrin	18.61 ± 0.01^f^	1.86	0.008	0.026
Quercitrin	19.07 ± 0.02^ef^	1.80	0.035	0.114
Quercetin	5.87 ± 0.01^a^	0.58	0.019	0.063
Kaempferol	5.36 ± 0.01^a^	0.53	0.026	0.085

Results are expressed as mean ± standard deviations (SD) of three determinations. Averages followed by different letters differ by Tukey's test at *P* < 0.001.
